# Performance of InSilicoVA for assigning causes of death to verbal autopsies: multisite validation study using clinical diagnostic gold standards

**DOI:** 10.1186/s12916-018-1039-1

**Published:** 2018-04-19

**Authors:** Abraham D. Flaxman, Jonathan C. Joseph, Christopher J. L. Murray, Ian Douglas Riley, Alan D. Lopez

**Affiliations:** 1 Institute for Health Metrics and Evaluation, University of Washington, Seattle, Washington USA; 2 School of Population and Global Health, University of Melbourne, Parkville, VIC Australia

**Keywords:** Verbal autopsy, Cause-of-death diagnosis, Validation

## Abstract

**Background:**

Recently, a new algorithm for automatic computer certification of verbal autopsy data named InSilicoVA was published. The authors presented their algorithm as a statistical method and assessed its performance using a single set of model predictors and one age group.

**Methods:**

We perform a standard procedure for analyzing the predictive accuracy of verbal autopsy classification methods using the same data and the publicly available implementation of the algorithm released by the authors. We extend the original analysis to include children and neonates, instead of only adults, and test accuracy using different sets of predictors, including the set used in the original paper and a set that matches the released software.

**Results:**

The population-level performance (i.e., predictive accuracy) of the algorithm varied from 2.1 to 37.6% when trained on data preprocessed similarly as in the original study. When trained on data that matched the software default format, the performance ranged from −11.5 to 17.5%. When using the default training data provided, the performance ranged from −59.4 to −38.5%. Overall, the InSilicoVA predictive accuracy was found to be 11.6–8.2 percentage points lower than that of an alternative algorithm. Additionally, the sensitivity for InSilicoVA was consistently lower than that for an alternative diagnostic algorithm (Tariff 2.0), although the specificity was comparable.

**Conclusions:**

The default format and training data provided by the software lead to results that are at best suboptimal, with poor cause-of-death predictive performance. This method is likely to generate erroneous cause of death predictions and, even if properly configured, is not as accurate as alternative automated diagnostic methods.

**Electronic supplementary material:**

The online version of this article (10.1186/s12916-018-1039-1) contains supplementary material, which is available to authorized users.

## Background

Reliable population-level cause-of-death estimates are critically important for designing effective public health policies [1]. Verbal autopsy (VA) is a key component of enhancing health information systems in many countries that do not have reliable civil registration and vital statistics systems [2, 3]. VA consists of a structured interview with family members of the deceased with the purpose of gathering enough information to infer the likely cause of death [4]. In some countries where up to 80–90% percent of deaths occur without medical attendance, VA provides the only usable information for generating population-level cause-of-death estimates with reasonable and representative coverage [5]. Computer algorithms that can reliably assign a cause of death greatly increase the feasibility of integrating VA routinely into civil registration and vital statistics (CRVS) systems. Computer certification of verbal autopsy (CCVA) allows systems to be scalable, consistent, and sustainable [6].

Numerous algorithms for predicting the cause of death from VAs have been developed over the last decade [7–11]. We previously developed a framework for validating the predictive accuracy of different diagnostic methods that allows for direct comparison of methods using the same standard set of criteria [12]. It provides a way of determining how well an algorithm will perform in different populations when the true distribution of causes of death is not known. This is crucial for generalizing results to new study populations and accurately capturing unknown changes in cause-of-death composition in the same population across time. We have used this procedure to determine the accuracy of a wide range of previously developed methods [13].

Recently, a new algorithm for CCVA called InSilicoVA was developed and published [14]. This method builds on previous research on the InterVA algorithm, and advances the approach by introducing an algorithm that quantifies uncertainty in the individual-level predictions and uses this information to better predict the cause distribution at the population level. This aligns well with the current global interest in using VA to estimate the distribution of causes of death for populations through routine application in vital registration systems. The authors use a range of metrics to determine the performance of their algorithm, including applying our assessment framework. However, the authors only validated the results for adult deaths and not child or neonatal deaths. Moreover, given the potential of such methods for transforming knowledge about cause-of-death patterns in populations for which little is currently known about the leading causes of death, we believe that an independent validation of their results is warranted before the method can be recommended for routine application.

In this study, we assess the diagnostic accuracy of the InSilicoVA algorithm for all ages using the same validation environment as used in the original InSilicoVA paper, namely the Population Health Metrics Research Consortium (PHMRC) gold standard database. We applied the validation procedure developed by Murray et al. [12] and assessed performance at the individual level, using chance-corrected concordance (CCC), and at the population level, using chance-corrected cause-specific mortality fraction (CCCSMF) accuracy.

## Methods

### Algorithm

InSilicoVA [14] is a Bayesian framework that improves upon InterVA [10] by using information about symptoms that are, and are not endorsed, to estimate probabilities for each cause of death in a way that is comparable across observations, and by estimating the individual-level and population-level predictions simultaneously. The model is estimated using Markov chain Monte Carlo (MCMC) simulations. To produce usable results, the algorithm must run a sufficient number of samples to ensure convergence. The authors have released their algorithm as an R package with a computationally intensive MCMC calculation implemented in Java through the rJava package. The algorithm utilizes a matrix of conditional probabilities between each cause and each symptom. These propensities, which the authors call the *probbase*, capture the user’s initial estimate of the relative likelihood of a symptom being endorsed for a given cause of death. These estimates can be derived from data or from expert judgement. The R package allows the user to input his own probbase file and also provides a default probbase based on the InterVA project. Open-source code (licensed under the GNU General Public License version 2) for the R implementation of InSilicoVA is freely available online.

### Data

We used the publicly available PHMRC gold standard database [15, 16] to validate the InSilicoVA algorithm. This dataset contains VAs matched to cause-of-death diagnoses from medical records, with variable confidence. Cases in the dataset were initially identified from deaths in hospitals where strict,predetermined diagnostic criteria were satisfied. This ensured that the true cause of death was known with greater certainty than is often the case for deaths recorded in vital registration systems, where diagnostic misclassification is typically estimated to range between 30 and 60% [17, 18]. After identifying cases, blinded VAs were collected using a modified version of the World Health Organization (WHO) VA instrument. This resulted in a validation database of 12,530 records for which the true cause of death was known with reasonable certainty, and for which a full VA interview had been conducted.

VAs were collected from six sites in four different countries: Andhra Pradesh, India; Bohol, Philippines; Dar es Salaam, Tanzania; Mexico City, Mexico; Pemba Island, Tanzania; and Uttar Pradesh, India between 2007 and 2010. The database includes deaths for 7841 adults, 2064 children, 1620 neonates, and 1005 stillbirths. Following practice from previous research, we used the most aggregated cause list with 34 adult causes, 21 child causes, and 6 neonate causes (including stillbirth) to assess the accuracy of cause-of-death predictions. These cause-of-death lists are shown in Additional file 1.

### Validation framework

In this study, we follow the recommendations of Murray et al. for validating VA diagnostic methods [12]. For this procedure, the validation dataset is randomly divided into a train fold containing 75% of the observations and a test fold containing the remaining 25% of observations. This is repeated 500 times, resulting in 500 test-train sets, each with a different subset of the original observations. For each test-train set, any given record appears in either the train set or the test set, but not both. The test set is then resampled to an uninformative Dirichlet distribution. This ensures that the cause compositions of the train and test sets are uncorrelated, which provides a more robust measure of performance (for example, it prevents a naive prediction algorithm from guessing an accurate population-level distribution without utilizing information at the individual level). Additionally, because the cause composition varies substantially across the 500 test-train splits, it ensures that the algorithm is tested on datasets with a wide variety of cause distributions and that performance estimates are not skewed by overfitting to the most common cause in the training data. To assess performance at the individual level, we use the median CCC across causes [12]. To assess performance at the population level, we use CCCSMF accuracy [19]. CCC for a single cause is calculated as:

$$ CC{C}_j=\frac{\frac{T{P}_j}{T{P}_j+F{N}_j}-\frac{1}{N}}{1-\frac{1}{N}} $$

where *TP*_*j*_ is the number of true positives for cause *j*, *TN*_*j*_ is the number of true negatives, and *N* is the number of causes. Values range between −1.0 and 1.0, where 1.0 indicates perfect ability to detect (i.e., diagnose) a cause, 0.0 indicates random guessing, and negative 1.0 indicates no ability to detect a cause. To create an overall metric of individual-level prediction accuracy, we use the mean of the cause-specific CCCs. Cause-specific mortality fraction (CSMF) accuracy is calculated as:

$$ \mathrm{CSMFAccuracy}=1-\frac{\sum_{j=1}^k\mid {\mathrm{CSMF}}_j^{\mathrm{true}}-{\mathrm{CSMF}}_j^{\mathrm{pred}}\mid }{2\left(1-\underset{j}{\min}\left({\mathrm{CSMF}}_j^{\mathrm{true}}\right)\right)} $$

where ${CSMF}_j^{true}$ is the true fraction for cause *j* and ${CSMF}_j^{pred}$ is the predicted fraction for cause *j*. This statistic can be corrected for chance (see Flaxman et al. [19]); we calculate the CCCSMF accuracy as:

$$ \mathrm{CCCSMFAccuracy}=\frac{\mathrm{CSMFAccuracy}-\left(1-{e}^{-1}\right)}{1-\left(1-{e}^{-1}\right)} $$

Similarly to CCC, perfect CCCSMF accuracy is attained at value 1.0, and values near 0.0 indicate that the diagnostic procedure being applied is essentially equivalent to random guessing.

### InSilicoVA validation

The InSilicoVA R package allows for a range of customizations to the inputs used to predict the cause of death. We validate the algorithm using three different configurations of inputs to assess its usability and performance. These configurations are obtained as follows: (1) using the built-in default training data, (2) training the algorithm with inputs that resemble the defaults, and (3) training the algorithm with inputs that do not resemble the defaults. Following the practice established in Murray et al. [12], we also conduct the analysis without predictors derived from questions related to previous contact with the health care system. This produces estimates of diagnostic accuracy that could be more appropriate for generalizing to community deaths where the decedents had no medical contact [16]. For each of the three configurations, we test all three age groups both with and without health care experience questions.

#### With default probbase

The default configuration assumes the input data matches the InterVA4 format with 245 symptoms. It uses the conditional probabilities from InterVA to predict one of 60 causes. With the default configuration, no ancillary training data is required. To validate the default configuration, we mapped the PHMRC database to the InterVA format, and then we used InSilicoVA to predict the cause of death. We then mapped the predicted causes to the PHMRC gold standard list. We compared these mapped predictions to the known underlying cause as listed in the PHMRC database to calculate performance. Since the algorithm was not trained empirically with this configuration, we used the entire validation dataset to test the predictive performance. However, it is still essential to test the algorithm on datasets with different cause compositions, so we repeated this process on 500 test datasets, each with a cause composition drawn from an uninformative Dirichlet distribution and samples drawn from the complete dataset with replacement according to this cause composition. The predicted causes included 36 adult causes of death, 20 child causes, and 7 neonate causes. Of the 245 symptom predictors used by InSilicoVA, the PHMRC dataset contained data for 123 adult symptoms, 69 child symptoms, and 62 symptoms for neonates.

#### With empirical probbase

Next, we assessed how InSilicoVA performed with training data that matched its expected inputs. For this assessment, we mapped the PHMRC database to the InterVA symptoms, and the “gold standard” causes were mapped to the predicted causes. For each of the 500 test-train splits, we used the train split to calculate the empirical probability of an InterVA symptom being endorsed, conditional on the mapped cause. This conditional probability matrix was used as the input probbase for the algorithm. The test split was resampled to a Dirichlet cause distribution, and the algorithm predicted a cause from the default set of causes.

#### With empirical probbase matching Tariff 2.0

Finally, we assessed how the algorithm performed with training data of a different format than the standard inputs. For this assessment, the PHMRC database was mapped to the set of symptoms used by the Tariff 2.0 algorithm [7]. The data was mapped to 171 adult symptoms, 86 child symptoms, and 110 neonate symptoms. For each of the 500 test-train splits, we used the train split to calculate the empirical probability of a Tariff 2.0 symptom being endorsed conditional on the original PHMRC gold standard cause. We then used this empirical probability matrix in the InSilicoVA algorithm to predict causes of death. As before, we predicted for data in the test split after it had been resampled to a Dirichlet cause distribution. Of the three assessments, this configuration should be the most favorable towards InSilicoVA since it avoids any possible discrepancies between definitions of the PHMRC causes and the default causes, and it provides more symptom predictors for the algorithm to use.

The InSilicoVA R package provides 10 hyperparameters which allow users to tune the estimation procedure. Except where specifically mentioned, we used the default value provided by the InSilicoVA packages. The validity of the results depends on the Monte Carlo experiment successfully converging to a stable result. We repeated each experiment using three times the default number of simulations and assessed the number of splits that converged and any differences in the results. Convergence was assessed using the Heidelberger and Welch test included with the R package. We used the extract.prob function provided by the InSilicoVA package in all training exercises.

## Results

Tables 1 and 2 show the algorithmic performance of InSilicoVA at the individual level and population level, respectively, using the default probbase, training the algorithm on data with the same causes and symptoms as the default probbase, and training the algorithm on data with different causes and symptoms. At both the individual and population levels, the configuration using the causes published with the dataset and the Tariff 2.0 symptoms performed best across all age groups regardless of whether health care experience (HCE) variables were included. These variables are intended to reflect the impact of the extent of contact with health services prior to death in terms of additional information that might improve diagnostic accuracy.

**Table 1:** Median chance-corrected concordance (%) for InSilicoVA and Tariff 2.0

		InSilicoVA(default probbase)		InSilicoVA(InterVA training)		InSilicoVA(Tariff 2.0 training)		Tariff 2.0	
		Median	95% UI	Median	95% UI	Median	95% UI	Median	95% UI
Adult	No HCE	16.1	(16.0, 16.2)	28.7	(28.3, 29.2)	28.5	(28.3, 28.7)	37.8	(37.6, 37.9)
	HCE	21.0	(20.9, 21.1)	33.0	(32.8, 33.3)	34.1	(33.9, 34.5)	50.5	(50.2, 50.7)
Child	No HCE	29.2	(29.0, 29.4)	35.8	(35.5, 36.3)	38.8	(38.4, 39.5)	44.6	(44.2, 45.0)
	HCE	29.4	(29.2, 29.6)	36.1	(35.7, 36.6)	38.4	(38.1, 39.0)	52.5	(52.1, 53.0)
Neonate	No HCE	19.2	(19.1, 19.4)	28.4	(27.9, 29.0)	37.8	(37.2, 38.3)	42.3	(41.9, 42.6)
	HCE	17.6	(17.3, 17.8)	28.9	(28.4, 30.0)	37.9	(37.3, 38.4)	45.1	(44.6, 45.4)

Table 1 shows the individual-level performance as the median value and uncertainty interval (UI) across 500 test-train splits using different probbase matrices for prediction, by age group, with and without health care experience (HCE) questions included. InSilicoVA was run without training using the default probbase, with an empirical probbase derived from training data mapped to the InterVA format, and with an empirical probbase derived from training data mapped to the Tariff 2.0 format. Previously published Tariff 2.0 results are shown for comparison

**Table 2:** Median chance-corrected cause-specific mortality fraction accuracy for InSilicoVA and Tariff 2.0

		InSilicoVA(default probbase)		InSilicoVA(InterVA training)		InSilicoVA(Tariff 2.0 training)		Tariff 2.0	
		Median	95% UI	Median	95% UI	Median	95% UI	Median	95% UI
Adult	No HCE	−59.4	(−61.7, −57.7)	4.3	(2.6, 5.4)	2.1	(0.5, 3.9)	23.1	(21.6, 24.3)
	HCE	−40.1	(−41.3, −38.7)	12.1	(10.5, 13.4)	13.9	(12.6, 15.5)	37.6	(36.5, 38.9)
Child	No HCE	−46.2	(−48.4, −43.6)	−10.4	(−13.5, −7.3)	22.3	(20.7, 23.9)	30.5	(28.4, 32.4)
	HCE	−42.7	(− 47.9, −37.4)	−11.5	(−13.1, −8.2)	22.4	(20.6, 23.8)	41.1	(39.2, 42.0)
Neonate	No HCE	−39.9	(−43.8, −32.1)	10.9	(4.9, 15.1)	37.6	(33.7, 40.8)	49.2	(47.4, 52.2)
	HCE	−38.5	(−43.9, −34.0)	17.5	(12.8, 22.9)	34.4	(30.9, 37.3)	53.1	(50.9, 55.1)

Table 2 shows the population-level performance as the median value and uncertainty interval (UI) across 500 test-train splits using different probbase matrices for prediction, by age group, with and without health care experience (HCE) questions included. InSilicoVA was run without training using the default probbase, with an empirical probbase derived from training data mapped to the InterVA format, and with an empirical probbase derived from training data mapped to the Tariff 2.0 format. Previously published Tariff 2.0 results are shown for comparison

At the individual level, InSilicoVA performed best for predicting the cause of death for child deaths. Without HCE variables, the median CCC for child VAs was 29.2% (UI 29.0%, 29.4%) using the default probbase, 35.8% (uncertainty interval (UI) 35.5%, 36.3%) when training the algorithm on the default cause list and symptoms, and 38.8% (UI 38.4%, 39.5%) when using the causes and symptoms which best matched the data. For adults and neonates, InSilicoVA performed substantially worse with the default probbase than with the Tariff 2.0 causes and symptoms. The CCC for adults was 16.1% (UI 16.0%, 16.2%) using the defaults and 28.5% (UI 28.3%, 28.7%) using Tariff 2.0 causes and symptoms. The CCC for neonates was 19.2% (UI 19.1%, 19.4%) using the defaults and 37.8% (UI 37.2%, 38.3%) using the Tariff 2.0 causes and symptoms. For adults, training the algorithm using the default causes and symptoms yielded diagnostic accuracy very similar to that resulting from using Tariff 2.0 causes and symptoms, 28.7% (UI 28.3%, 29.2%) compared to 28.5% (UI 28.3%, 28.7%). For neonates, training using default symptoms and causes produced lower CCC, 28.4% (UI 27.9%, 29.0%) compared to 37.8% (UI 37.2%, 38.3%) when training using the Tariff 2.0 symptoms. The cause-specific performance, as measured by CCC varied significantly by cause. Tables 3, 4, and 5 summarize the cause-specific CCC. When the model was trained using Tariff 2.0 symptoms, the adult causes with the highest CCC were Bite of venomous animal, Drowning, Maternal; the child causes with the highest CCC were Bite of venomous animal, Drowning, Road traffic; and the neonate causes with the highest CCC were Meningitis/sepsis, Preterm delivery, Stillbirth. Across all age groups, seven causes were predicted at or below the level of random guessing. Additional files 2, 3, and 4 present the full misclassification matrix for cause-specific performance of InSilicoVA when trained using Tariff 2.0 symptoms, and predicted without health care experience, to show the detailed patterns of prediction at the individual level.

**Table 3:** Median adult cause-specific chance-corrected concordance (%) for InSilicoVA and Tariff 2.0

		InSilicoVA (Tariff 2.0 training)				Tariff 2.0			
		No HCE		HCE		No HCE		HCE	
PHMRC cause	ICD10	Median	95% UI	Median	95% UI	Median	95% UI	Median	95% UI
Diarrhea/dysentery	A09	18.0	(16.8, 19.1)	21.5	(20.5, 22.5)	36.4	(35.1, 36.7)	38.5	(37.8, 39.3)
Tuberculosis	A16	23.2	(22.5, 24.7)	34.4	(32.8, 36.0)	42.2	(41.8, 43.1)	43.5	(43.1, 44.3)
AIDS	B24	13.2	(11.7, 14.9)	30.2	(29.1, 31.3)	44.3	(43.7, 45.1)	51.0	(50.5, 51.8)
Malaria	B54	30.1	(27.5, 31.3)	33.5	(31.3, 35.9)	42.3	(42.3, 43.8)	57.9	(55.2, 58.8)
Other infectious diseases	B99	3.5	(2.7, 4.4)	7.7	(6.6, 9.0)	6.3	(6.3, 6.7)	15.9	(15.5, 16.6)
Esophageal cancer	C15	61.5	(59.5, 63.6)	61.4	(58.8, 63.2)	69.1	(61.4, 69.1)	79.4	(79.4, 79.4)
Stomach cancer	C16	27.2	(25.2, 29.2)	26.3	(25.5, 28.4)	16.3	(16.3, 16.3)	29.2	(29.2, 35.6)
Colorectal cancer	C18	0.9	(−0.3, 1.9)	1.7	(1.0, 3.0)	5.9	(5.2, 9.3)	17.6	(17.6, 17.6)
Lung cancer	C34	35.9	(34.0, 37.4)	41.7	(38.9, 43.7)	32.6	(32.6, 34.1)	28.7	(28.7, 28.7)
Breast cancer	C50	40.4	(39.4, 42.0)	48.5	(47.4, 50.9)	69.8	(68.5, 70.6)	74.8	(74.8, 76.4)
Cervical cancer	C53	60.4	(58.8, 61.6)	65.7	(64.1, 67.7)	70.9	(70.1, 70.9)	75.8	(73.6, 76.2)
Prostate cancer	C61	31.8	(30.0, 33.9)	36.3	(33.3, 38.2)	48.5	(48.5, 48.5)	65.7	(62.5, 65.7)
Leukemia/lymphomas	C96	7.2	(5.9, 8.7)	16.6	(15.1, 17.6)	28.7	(26.8, 28.7)	34.9	(34.0, 36.6)
Diabetes	E14	13.0	(12.3, 14.5)	24.7	(23.5, 26.2)	46.9	(46.2, 47.7)	50.9	(50.2, 51.6)
Epilepsy	G40	16.2	(14.1, 18.0)	29.6	(26.5, 32.4)	48.5	(48.5, 48.5)	57.1	(57.1, 57.1)
Acute myocardial infarction	I21	27.1	(25.3, 28.7)	31.4	(31.2, 33.0)	39.6	(38.9, 40.2)	44.4	(43.5, 44.9)
Stroke	I64	43.6	(41.9, 44.5)	52.1	(51.1, 53.9)	47.1	(46.5, 47.7)	50.4	(49.8, 51.0)
Other cardiovascular diseases	I99	12.6	(11.6, 13.6)	19.4	(18.1, 20.5)	30.7	(30.3, 31.3)	37.3	(36.4, 38.0)
Pneumonia	J22	−0.5	(−0.8, 0.1)	8.4	(7.3, 9.5)	5.5	(5.2, 5.8)	15.2	(14.7, 15.5)
COPD	J33	25.6	(24.5, 27.3)	36.2	(34.1, 38.1)	38.2	(37.7, 39.6)	40.1	(38.7, 40.1)
Asthma	J45	9.6	(7.0, 11.6)	15.6	(14.1, 18.0)	58.8	(57.1, 65.7)	57.1	(57.1, 65.7)
Cirrhosis	K74	23.9	(22.6, 25.5)	39.1	(38.0, 40.5)	25.1	(24.4, 25.8)	51.2	(50.5, 51.9)
Renal failure	N19	−3.0	(−3.0, −3.0)	0.4	(−0.3, 1.4)	9.8	(9.5, 10.2)	28.9	(28.5, 29.6)
Maternal	O95	74.2	(72.2, 75.5)	77.1	(75.6, 78.2)	68.3	(67.5, 69.1)	68.0	(67.3, 68.4)
Other non-communicable diseases	R100	−3.0	(−3.0, −3.0)	−0.5	(−1.1, 0.2)	11.7	(11.3, 12.0)	14.6	(14.1, 15.0)
Road traffic	V89	36.9	(34.3, 38.1)	38.2	(36.3, 39.6)	77.3	(77.1, 78.5)	81.5	(81.5, 82.5)
Falls	W19	29.5	(27.6, 31.2)	34.1	(31.3, 35.5)	57.2	(56.9, 58.3)	59.3	(58.8, 60.4)
Drowning	W74	86.7	(85.3, 88.5)	82.7	(81.3, 83.3)	84.1	(83.5, 84.1)	81.3	(80.2, 84.1)
Fires	X09	24.0	(22.2, 26.2)	26.9	(25.1, 28.7)	70.6	(69.1, 72.5)	71.7	(69.1, 72.5)
Bite of venomous animal	X27	72.9	(70.1, 74.8)	74.2	(72.3, 76.1)	87.1	(87.1, 87.1)	80.7	(80.7, 80.7)
Poisonings	X49	21.2	(19.0, 22.7)	21.0	(19.4, 23.3)	34.4	(34.4, 34.4)	57.9	(55.8, 57.9)
Other injuries	X58	55.3	(52.4, 57.1)	65.6	(62.7, 66.3)	68.3	(68.3, 68.3)	72.3	(69.1, 72.3)
Suicide	X84	13.6	(12.4, 15.2)	19.1	(17.5, 20.5)	7.5	(6.9, 9.8)	9.8	(7.6, 10.3)
Homicide	Y09	26.6	(25.2, 27.9)	31.2	(29.5, 32.6)	73.0	(72.2, 73.0)	78.4	(77.9, 79.9)

Table 3 shows the cause-specific chance-corrected concordance for adult data for InSilicoVA using an empirical probbase derived from training data mapped to the Tariff 2.0 format. Previously published Tariff 2.0 results are shown for comparison

*PHMRC* Population Health Metrics Research Consortium, *ICD* International Classification of Diseases, *HCE* health care experience, *UI* uncertainty interval, *COPD* chronic obstructive pulmonary disease

**Table 4:** Median child cause-specific chance-corrected concordance (%) for InSilicoVA and Tariff 2.0

		InSilicoVA (Tariff 2.0 training)				Tariff 2.0			
		No HCE		HCE		No HCE		HCE	
PHMRC cause	ICD10	Median	95% UI	Median	95% UI	Median	95% UI	Median	95% UI
Diarrhea/dysentery	A09	16.0	(14.1, 18.0)	15.1	(12.5, 16.7)	24.6	(24.0, 25.5)	37.0	(36.3, 38.2)
Sepsis	A41	−2.8	(−5.0, −1.5)	−5.0	(−5.0, −2.7)	12.5	(11.2, 13.5)	14.7	(14.1, 16.0)
Hemorrhagic fever	A99	16.0	(14.5, 19.0)	16.0	(13.6, 19.7)	51.5	(51.5, 52.3)	59.6	(59.6, 65.0)
Measles	B05	73.8	(70.2, 77.2)	71.9	(67.8, 73.8)	82.5	(82.5, 82.5)	82.5	(82.5, 82.5)
AIDS	B24	42.7	(39.1, 47.4)	42.1	(38.2, 47.4)	37.0	(37.0, 37.0)	58.0	(58.0, 58.0)
Malaria	B54	47.5	(47.2, 49.8)	49.3	(47.4, 51.3)	41.7	(40.0, 42.1)	57.2	(56.6, 58.8)
Other infectious diseases	B99	24.8	(22.8, 27.0)	25.0	(21.4, 27.2)	7.4	(7.4, 10.0)	26.5	(25.9, 27.8)
Other cancers	C76	4.3	(−2.8, 6.2)	5.0	(1.8, 8.0)	25.0	(25.0, 30.0)	40.0	(40.0, 40.0)
Meningitis	G03	15.0	(12.5, 16.4)	13.3	(11.9, 15.5)	27.3	(25.0, 32.5)	30.0	(26.5, 32.5)
Encephalitis	G04	29.9	(26.5, 32.2)	29.8	(24.8, 33.0)	37.0	(37.0, 37.0)	41.7	(37.0, 47.5)
Other cardiovascular diseases	I99	7.7	(6.1, 10.4)	10.8	(8.9, 13.9)	11.6	(11.6, 12.5)	33.7	(33.7, 33.7)
Pneumonia	J22	16.8	(15.6, 18.3)	16.0	(14.4, 17.5)	7.7	(7.1, 8.1)	9.9	(9.1, 10.8)
Other digestive diseases	K92	4.0	(2.4, 5.5)	4.2	(2.3, 6.7)	3.8	(3.8, 3.8)	21.3	(21.3, 21.3)
Other defined causes of child deaths	R101	13.0	(11.2, 14.5)	11.2	(8.8, 12.3)	5.5	(4.5, 6.2)	16.9	(16.0, 18.3)
Road traffic	V89	90.1	(88.8, 91.2)	89.5	(88.3, 91.2)	90.5	(88.9, 90.9)	90.9	(90.9, 92.5)
Falls	W19	54.6	(51.7, 57.6)	55.9	(52.9, 57.9)	73.8	(73.8, 73.8)	73.8	(73.8, 73.8)
Drowning	W74	92.5	(90.7, 93.6)	92.5	(91.2, 94.1)	90.0	(89.5, 90.0)	93.8	(92.5, 94.8)
Fires	X09	50.9	(47.5, 52.9)	48.4	(47.4, 52.2)	69.1	(69.1, 69.1)	75.3	(73.8, 75.3)
Bite of venomous animal	X27	88.5	(86.8, 90.4)	87.7	(85.6, 89.5)	92.5	(92.5, 92.5)	92.5	(92.5, 100.0)
Poisonings	X49	37.1	(30.0, 42.3)	40.0	(34.4, 45.3)	47.5	(47.5, 47.5)	73.8	(73.8, 73.8)
Violent death	Y09	75.3	(73.7, 76.8)	76.6	(73.7, 79.0)	83.8	(82.5, 83.8)	83.8	(83.8, 83.8)

Table 4 shows the cause-specific chance-corrected concordance for child data for InSilicoVA using an empirical probbase derived from training data mapped to the Tariff 2.0 format. Previously published Tariff 2.0 results are shown for comparison

*PHMRC* Population Health Metrics Research Consortium, *ICD* International Classification of Diseases, *HCE* health care experience, *UI* uncertainty interval

**Table 5:** Median neonate cause-specific chance-corrected concordance (%) for InSilicoVA and Tariff 2.0

		InSilicoVA (Tariff 2.0 training)				Tariff 2.0			
		No HCE		HCE		No HCE		HCE	
PHMRC cause	ICD10	Median	95% UI	Median	95% UI	Median	95% UI	Median	95% UI
Pneumonia	J22	5.6	(4.0, 8.0)	3.5	(1.6, 5.8)	31.4	(31.4, 37.1)	37.1	(37.1, 37.1)
Preterm delivery	P07	50.5	(49.3, 52.0)	52.0	(50.7, 53.3)	41.5	(40.8, 42.2)	40.5	(40.0, 41.1)
Birth asphyxia	P21	38.0	(36.7, 39.5)	38.7	(37.1, 40.0)	22.0	(21.4, 22.6)	25.9	(25.1, 26.6)
Meningitis/sepsis	P36	43.8	(41.6, 45.5)	44.3	(42.9, 46.0)	37.9	(36.9, 38.8)	44.3	(43.2, 45.1)
Stillbirth	P95	90.9	(90.3, 91.2)	91.0	(90.6, 91.4)	85.7	(85.1, 86.2)	85.6	(85.1, 86.1)
Congenital malformation	Q89	−0.9	(−1.6, 0.4)	−1.0	(−1.9, 0.3)	34.2	(32.9, 34.2)	34.8	(34.2, 36.1)

Table 5 shows the cause-specific chance-corrected concordance for neonate data for InSilicoVA using an empirical probbase derived from training data mapped to the Tariff 2.0 format. Previously published Tariff 2.0 results are shown for comparison

*PHMRC* Population Health Metrics Research Consortium, *ICD* International Classification of Diseases, *HCE* health care experience, *UI* uncertainty interval

At the population level, InSilicoVA performed best in predicting the CSMF for neonates when provided with training data. The algorithm performed substantially worse than chance for all age groups using the default probbase, despite predicting better than chance at the individual level for adults and children. The median CCCSMF was −59.4% (UI –61.7%, −57.7%) for adults, −46.2% (UI –48.4%, −43.6%) for children, and −39.9% (UI –43.8%, −32.1%) for neonates. The median CCCSMF was higher for child and neonate age groups when using the Tariff 2.0 causes and symptoms. For adults, the performance was the same when using the InterVA or Tariff 2.0 training. The CCCSMF was 2.1% (UI 0.5%, 3.9%) for adults, 22.3% (UI 20.7%, 23.9%) for children, and 37.6% (UI 33.7%, 40.8%) for neonates.

**Fig. 1:**
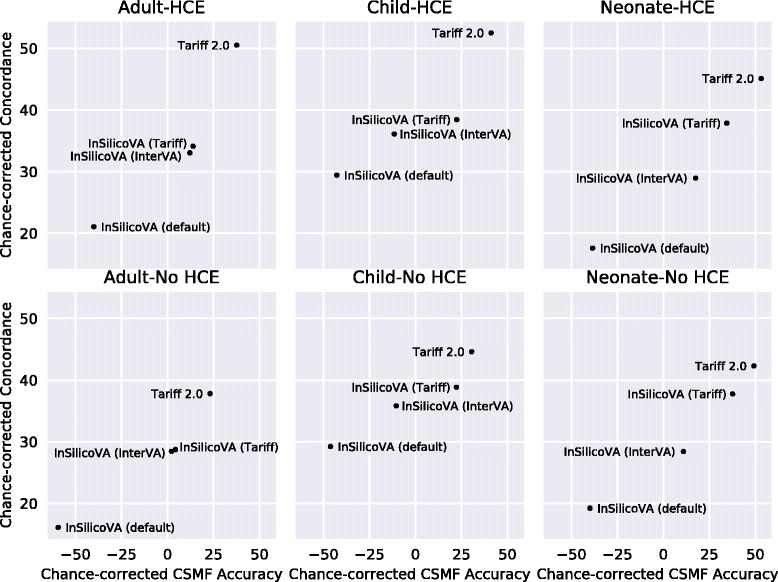
Comparison of InSilicoVA and Tariff 2.0 at the individual and population levels. Note: Individual-level accuracy is assessed using chance-corrected concordance. Population-level accuracy is assessed using chance-corrected cause-specific mortality fraction (CSMF) accuracy. Values of zero in either dimension are equivalent to random guessing and range up to 100% for perfect accuracy. InSilicoVA is tested using the default expert-derived probbase, a probbase empirically trained using InterVA symptoms, and a probbase empirically trained using Tariff 2.0 symptoms. Published accuracies of Tariff 2.0 are shown for comparison

At both the individual level and the population level, Tariff 2.0 outperformed InSilicoVA in all age groups. At the individual level without HCE variables, the median CCC across splits was 9.3 percentage points higher for adults, 5.8 percentage points higher for children, and 4.5 percentage points higher for neonates using Tariff 2.0 to diagnose the VAs, compared to InSilicoVA. At the population level, the median CCCSMF for Tariff 2.0 was 21.0 percentage points higher for adults, 8.2 percentage points higher for children, and 11.6 percentage points higher for neonates. Figure 1 shows the individual-level and population-level performance of InSilicoVA using different configurations compared to Tariff 2.0. The cause-specific performance of InSilicoVA tended to follow a similar pattern as the Tariff 2.0 algorithm when trained using the same symptoms as predictors, except that the Tariff 2.0 concordance was generally higher. Across the specific age groups, InSilicoVA had higher concordance only for Drowning, Lung cancer, Maternal, Stomach cancer, and Suicide in adults; AIDS, Drowning, Malaria, Other defined causes of child deaths, Other digestive diseases, Other infectious diseases, and Pneumonia in children; and Birth asphyxia, Meningitis/sepsis, Preterm delivery, and Stillbirth in neonates.

Across all age groups, InSilicoVA had higher sensitivity for 22 of 61 PHMRC causes for at least one of the with HCE/without HCE scenarios. It had higher specificity for 32 of 61 PHMRC causes. Table 6 shows the median sensitivity and specificity across cause for InSilicoVA and Tariff 2.0. Overall Tariff 2.0 had higher sensitivity for all age groups with and without the health care experience predictors. InSilicoVA had comparable specificity to Tariff 2.0 for adults and children, but slightly lower specific for neonates. Additional file 5 shows cause-specific comparisons of InSilicoVA and Tariff 2.0 using sensitivity and specificity.

**Table 6:** Median sensitivity and specificity (%) for InSilicoVA and Tariff 2.0

		InSilicoVA (Tariff 2.0 training)		Tariff 2.0	
		Median sensitivity	Median specificity	Median sensitivity	Median specificity
Adult	No HCE	30.4	98.1	39.8	98.2
	HCE	35.9	98.2	52.1	98.5
Child	No HCE	41.0	97.7	46.7	97.4
	HCE	40.9	97.6	55.2	97.7
Neonate	No HCE	48.4	90.4	53.9	93.5
	HCE	48.5	90.2	56.3	93.8

Table 6 shows the sensitivity and specificity across causes for InSilicoVA using an empirical probbase derived from training data mapped to the Tariff 2.0 format. Previously published Tariff 2.0 results are shown for comparison

Further, when using training data, the model did not always converge for every test-train split. Across the three modules and different mappings of training data, for 81.5–4.7% of the 500 test-train splits the model did not converge when using the default number of Monte Carlo simulations. We increased the number of simulations performed during the fitting process to three times the default to see if the model would eventually converge. Even with these extra samples, up to 27.8% of splits still failed to converge for some configurations.

## Discussion

As expected, InSilicoVA performed best when using the causes and symptoms that closely matched the data. The differences between using the causes and symptoms from the data versus mapping to the InterVA causes and symptoms were greatest for neonates. The differences in population-level accuracy were generally larger than at the individual level. Even when using the ideal configuration, InSilicoVA always had lower diagnostic accuracy than the Tariff 2.0 method. The difference was greatest for adults where, without health care variables, the predictive accuracy of InSilicoVA was 9.3 percentage points lower at the individual level and 21.0 percentage points lower at the population level. This poorer performance, particularly for adult deaths, has significant implications for estimating cause-of-death patterns in countries where, in all cases, the vast majority of deaths occur among the adult population [20].

We have reviewed InSilicoVA for two complementary purposes. First, we assessed the performance of the InSilicoVA method as a diagnostic algorithm for verbal autopsy. Second, InSilicoVA is a new piece of software that potentially could be applied routinely into vital statistics systems for deaths without physician certification. Knowing that this is a potential use for this software, it is obviously important that the method can be easily applied, and with confidence about diagnostic accuracy, in settings with little technical and statistical support. The need for continuous vetting of model input parameters and verification of model convergence is likely to be problematic in many countries, and is likely to result in low-quality cause-of-death statistics in countries where there are insufficient resources to procure these services.

Compared with Tariff 2.0, we found that InSilicoVA performs significantly worse in correctly predicting causes of death. We were not able to identify any configuration of input parameters, for any age group, that outperformed published estimates from the Tariff 2.0 algorithm. InSilicoVA shows the most promising results for child and neonates, despite having noticeably fewer symptom predictors for these age groups, but even for these age groups it still has noticeably lower diagnostic accuracy than Tariff 2.0. This result is generally consistent when comparing cause-specific performance between the two algorithms. For a few causes, InSilicoVA had higher CCC. However, the increased sensitivity was at the expense of other causes, which had significantly lower concordance and may indicate that the model overfits to causes which may be easier to detect. This is especially evident for the neonate, where InSilicoVA achieved higher concordance for four causes, but predicted the other two causes at level equal to chance, as indicated by the uncertainty interval containing zero. This is in contrast to Tariff 2.0, which performed similarly across causes, with the exception of Stillbirth, which had high concordance for both algorithms.

To predict with this algorithm, users must decide what conditional probability matrix to use. The InSilicoVA authors propose that, in practice, ranked conditional probabilities be derived from expert panels that rank the propensities of seeing a symptom given a particular cause of death [14]. They show that the predictive accuracy of the method is heavily dependent on the quality of this input. However, deriving these probabilities may not be straightforward. The required value is the probability of a *respondent saying* the decedent had a given symptom. This is subtly but importantly different from the probability of the *decedent having* the symptom. The value needed for this algorithm requires that a decedent had a symptom, the decedent communicates this symptom to someone or someone notices it, the interviewer finds this person who knew about the symptom, and the respondent remembers the symptom months later when the VA interview is being conducted. The respondent may not notice or may forget key symptoms. When medical professionals create these ranked conditional probabilities, they may implicitly estimate the probability of identifying a symptom themselves in their expert, clinical evaluation. This value could mislead the algorithm and result in inaccurate predictions. It is necessary that experts who select these conditional probabilities balance both the presentation of symptoms due to a disease and the ability of non-experts to reliably identify, remember, and report on these symptoms.

We report here, for the first time, the predictive performance of InSilicoVA using the default conditional probabilities (from InterVA). Given resource constraints in the settings where VA is likely to be used, and the logistical difficulties of collecting location-specific probbase information from medical professionals familiar with the area, it is quite likely that the InSilicoVA defaults will be used in practice. We found that the default configuration and conditional probabilities consistently perform worse than chance at all ages at the population level. The authors claim that InSilicoVA is applicable in a wider range of settings because it does not need to rely on “gold standard” data [14]. However, we have demonstrated that using expert-derived training as opposed to empirically derived training data results in unacceptably poor performance.

The results from this study match a previous validation of the InterVA algorithm, which found that, once corrected for chance, population-level accuracy of predictions using an expert-derived probbase are relatively poor [21]. The InterVA probbase used by InSilicoVA has undergone extensive field testing and review by numerous investigators in multiple countries [22]. Given this, we believe it is extremely unlikely for expert-derived probbases to produce estimates that rival empirically derived training such as that used by Tariff 2.0. Additionally, expert-derived training has the unfortunate effect of often appearing plausible, since it reconfirms the intuition of the experts training and evaluating the method, which can be, and often is, incorrect. The net result is a situation in which diagnostic information being provided by InSilicoVA is likely to be worse than acting on no information whatsoever.

In this study, we used test data with a cause distribution uncorrelated with the training data. This resulted in scenarios in which the training data and test data were sufficiently different that the model could not successfully converge. The R package displays a warning about non-convergence and says the results may be unreliable, but it still yields outputs. This raises two operational considerations with the use of InSilicoVA. First, it is possible to create a conditional probability matrix in which the model does not successfully produce reliable results. Second, the R package produces results even in this circumstance. It is possible that InSilicoVA users may unintentionally overlook the warning that the MCMC process has not converged, leading to adoption of results which are known to be statistically inaccurate.

Installing Java and properly configuring R and Java to work together requires considerable technical expertise and is not standardized across different computer systems. Although InSilicoVA is freely available, it may require expert technical consultation to be usable.

## Conclusions

Verbal autopsy as a diagnostic method is now being actively considered by countries for routine widespread use in surveillance and vital statistics systems [23]. It is important to keep improving the science behind estimation and validation of different cause-of-death prediction strategies so that policy makers can be provided with the highest quality estimates based on the best possible measurement methods. It is also important that methods be independently investigated and evaluated for usability for governments in low- and middle-income countries seeking to reduce ignorance about who dies of what.

The InSilicoVA algorithm provides some key advances in CCVA. Unlike previous algorithms, it provides a method for calculating the uncertainty in each prediction. However, implementing the algorithm effectively requires both an increased level of technical expertise to utilize R and Java and conceptual expertise to tune model hyperparameters and interpret convergence from a hierarchical Bayesian model. Additionally, our results indicate that the default setting for conditional probabilities that come with the R package is suboptimal. This means that users should be very cautious about applying this new method.

Moreover, in the validation environment we have defined using the PHMRC database, InSilicoVA was found to be less accurate than Tariff 2.0 in predicting both causes of death for individuals (by about 10% in CCC) and the cause of death distribution in a population (about 20% less accurate in CCCSMF), with the differences being more marked for adult and child deaths than for neonates. For 20 out of 61 causes of death, InSilicoVA was found to have higher sensitivity than Tariff 2.0 (when both were run without health care experience), while for 40 the sensitivity was lower. Since the vast majority of deaths in low- and middle-income countries now occur among children and adults, rather than neonates, the higher CCCSMF accuracy of Tariff 2.0 in predicting causes of death, along with its ease of application, should make it the method of choice for countries seeking to maximize the accuracy and cost-effectiveness of automated verbal autopsy in their national CRVS systems.

## Additional files

Additional file 1: Gold standard causes. Causes from the PHMRC gold standard database and mapping to causes used by InSilicoVA by age group. (DOCX 11 kb)

Additional file 2: Misclassification matrix for adult deaths when InSilicoVA is trained using Tariff 2.0 symptoms without health care experience questions. (XLSX 11 kb)

Additional file 3: Misclassification matrix for child deaths when InSilicoVA is trained using Tariff 2.0 symptoms without health care experience questions. (XLSX 7 kb)

Additional file 4: Misclassification matrix for neonatal deaths when InSilicoVA is trained using Tariff 2.0 symptoms without health care experience questions. (XLSX 5 kb)

Additional file 5: Cause-specific sensitivity and specificity for InSilicoVA and Tariff 2.0. (XLSX 13 kb)

## Abbreviations

CCC: Chance-corrected concordance
CCCSMF accuracy: Chance-corrected cause-specific mortality fraction accuracy
CCVA: Computer certification of verbal autopsy
CRVS: Civil registration and vital statistics
CSMF: Cause-specific mortality fraction
MCMC: Markov chain Monte Carlo
PHMRC: Population Health Metrics Research Consortium
VA: Verbal autopsy
