# Supplementary material for: Performance of InSilicoVA for assigning causes of death to verbal autopsies: multisite validation study using clinical diagnostic gold standards
Source: BMC Med. 2018 Apr 19;16:56. doi: 10.1186/s12916-018-1039-1 (PMC5907465; doi:10.1186/s12916-018-1039-1)
Supplement: Supplementary file 1 — Gold standard causes. Causes from the PHMRC gold standard database and mapping to causes used by InSilicoVA by age group. (DOCX 11 kb) [file 12916_2018_1039_MOESM1_ESM.docx]

# Additional file 1

List of causes of death from the Population Health Metric Research Consortium database and mapping to causes used by InsilicoVA.

## Adult causes

The more detailed list of 46 causes was used to map PHMRC causes to the InSilicoVA causes. This provides better matching of some causes, especially for maternal causes. Analyses using PHMRC causes used the more aggregated list of 34 causes.

| ICD 10 | PHMRC Cause 46 | PHMRC Cause 34 | InSilicoVA Cause |
| --- | --- | --- | --- |
| A09 | Diarrhea/Dysentery | Diarrhea/Dysentery | Diarrhoeal diseases |
| A16 | TB | TB | Pulmonary tuberculosis |
| B20 | AIDS with TB | AIDS | HIV/AIDS related death |
| B24 | AIDS | AIDS | HIV/AIDS related death |
| B54 | Malaria | Malaria | Malaria |
| B99 | Other Infectious Diseases | Other Infectious Diseases | Other and unspecified infect dis |
| C15 | Esophageal Cancer | Esophageal Cancer | Digestive neoplasms |
| C16 | Stomach Cancer | Stomach Cancer | Digestive neoplasms |
| C18 | Colorectal Cancer | Colorectal Cancer | Other and unspecified neoplasms |
| C34 | Lung Cancer | Lung Cancer | Respiratory neoplasms |
| C50 | Breast Cancer | Breast Cancer | Breast neoplasms |
| C53 | Cervical Cancer | Cervical Cancer | Reproductive neoplasms MF |
| C61 | Prostate Cancer | Prostate Cancer | Reproductive neoplasms MF |
| C80 | Other Cancers | Other Non-communicable Diseases | Other and unspecified neoplasms |
| C94 | Leukemia | Leukemia/Lymphomas | Other and unspecified neoplasms |
| C96 | Lymphomas | Leukemia/Lymphomas | Other and unspecified neoplasms |
| E11 | Diabetes with Skin Infection/Sepsis | Diabetes | Diabetes mellitus |
| E11 | Diabetes with Renal Failure | Diabetes | Diabetes mellitus |
| E11 | Diabetes with Coma | Diabetes | Diabetes mellitus |
| G40 | Epilepsy | Epilepsy | Epilepsy |
| I21 | Acute Myocardial Infarction | Acute Myocardial Infarction | Acute cardiac disease |
| I50 | Congestive Heart Failure | Other Cardiovascular Diseases | Other and unspecified cardiac dis |
| I52 | Inflammatory Heart Disease | Other Cardiovascular Diseases | Acute cardiac disease |
| I64 | Stroke | Stroke | Stroke |
| I99 | Other Cardiovascular Diseases | Other Cardiovascular Diseases | Other and unspecified cardiac dis |
| J22 | Pneumonia | Pneumonia | Acute resp infect incl pneumonia |
| J33 | COPD | COPD | Chronic obstructive pulmonary dis |
| J45 | Asthma | Asthma | Asthma |
| K74 | Cirrhosis | Cirrhosis | Liver cirrhosis |
| K92 | Other Digestive Diseases | Other Non-communicable Diseases | Acute abdomen |
| N19 | Renal Failure | Renal Failure | Renal failure |
| O16 | Hypertensive Disorder | Maternal | Pregnancy-induced hypertension |
| O23 | Sepsis | Maternal | Pregnancy-related sepsis |
| O67 | Hemorrhage | Maternal | Obstetric haemorrhage |
| O95 | Other Pregnancy-Related Deaths | Maternal | Other and unspecified maternal CoD |
| O99 | Anemia | Maternal | Anaemia of pregnancy |
| R100 | Other Non-communicable Diseases | Other Non-communicable Diseases | Other and unspecified NCD |
| V89 | Road Traffic | Road Traffic | Road traffic accident |
| W19 | Falls | Falls | Accid fall |
| W74 | Drowning | Drowning | Accid drowning and submersion |
| X09 | Fires | Fires | Accid expos to smoke fire & flame |
| X27 | Bite of Venomous Animal | Bite of Venomous Animal | Contact with venomous plant/animal |
| X49 | Poisonings | Poisonings | Accid poisoning & noxious subs |
| X58 | Other Injuries | Other Injuries | Other and unspecified external CoD |
| X84 | Suicide | Suicide | Intentional self-harm |
| Y09 | Homicide | Homicide | Assault |

## Child causes

| ICD 10 | PHMRC Cause | InSilicoVA Cause |
| --- | --- | --- |
| A09 | Diarrhea/Dysentery | Diarrhoeal diseases |
| A41 | Sepsis | Sepsis (non-obstetric) |
| A99 | Hemorrhagic fever | Haemorrhagic fever |
| B05 | Measles | Measles |
| B24 | AIDS | HIV/AIDS related death |
| B54 | Malaria | Malaria |
| B99 | Other Infectious Diseases | Other and unspecified infect dis |
| C76 | Other Cancers | Other and unspecified neoplasms |
| G03 | Meningitis | Meningitis and encephalitis |
| G04 | Encephalitis | Meningitis and encephalitis |
| I99 | Other Cardiovascular Diseases | Other and unspecified cardiac dis |
| J22 | Pneumonia | Acute resp infect incl pneumonia |
| K92 | Other Digestive Diseases | Acute abdomen |
| R101 | Other Defined Causes of Child Deaths | Other and unspecified NCD |
| V89 | Road Traffic | Road traffic accident |
| W19 | Falls | Accid fall |
| W74 | Drowning | Accid drowning and submersion |
| X09 | Fires | Accid expos to smoke fire & flame |
| X27 | Bite of Venomous Animal | Contact with venomous plant/animal |
| X49 | Poisonings | Accid poisoning & noxious subs |
| Y09 | Violent Death | Assault |

## Neonate causes

| ICD 10 | PHMRC Cause | InSilicoVA Cause |
| --- | --- | --- |
| J22 | Pneumonia | Neonatal pneumonia |
| P07 | Preterm Delivery (with or without RDS) and Sepsis | Prematurity |
| P07 | Preterm Delivery (without RDS) and Sepsis and Birth Asphyxia | Prematurity |
| P07 | Preterm Delivery (without RDS) and Birth Asphyxia | Prematurity |
| P07 | Preterm Delivery without Respiratory Distress Syndrome | Prematurity |
| P21 | Birth asphyxia | Birth asphyxia |
| P22 | Preterm Delivery with Respiratory Distress Syndrome | Prematurity |
| P36 | Meningitis/Sepsis | Meningitis and encephalitis |
| P39 | Sepsis with Local Bacterial Infection | Neonatal sepsis |
| P95 | Stillbirth | Fresh stillbirth |
| Q89 | Congenital malformation | Congenital malformation |
